# S1-4 Decisive factors for movement in Danish schools

**DOI:** 10.1093/eurpub/ckad133.007

**Published:** 2023-09-11

**Authors:** Jacob Have Neilson

**Affiliations:** Dansk Skoleidræt

## Abstract

**Purpose:**

What is needed to succeed with implementation of movement as a natural part of the school day? This question has often been asked to different stakeholders in the Danish school system.

**Methods:**

Based on a systemic approach, the Practice Center for School Sport and Movement conducted a Delphi study inviting stakeholders from all sectors of the system in order to identify important factors based on dialogue and common understanding across sectors. 57 practitioners representing all sectors in the Danish school system from national politicians to pupils participated in the process of identifying decisive factors for movement in Danish schools.

449 inputs were identified from three different sources. 1) Literature review, 2) Experiences from experts working with promoting implementation of movement in Danish schools and 3) Experiences from practitioners collected via surveys distributed through Social Media channels.

**Results:**

The 449 inputs resulted in 169 factors which the expert panel rated in an online survey. Consequently 136 factors qualified for further discussion. The 136 factors were distributed in 12 areas reflecting the sectors of the school system, and panelists were asked to rank factors within an area from ‘most important’ to ‘least important’ in a pyramid model. Panelists wrote arguments for factors placed as most and least important, which served as feedback supporting the dialogue in the two following workshops where they in groups across sectors discussed and prioritized factors within each area.

The panel identified common understanding of movement as a didactical tool which supports variation in school day activities. Furthermore, the panel pointed at priority in terms of time to create culture, structures which support culture and resource persons or teams who support colleagues in development of competencies. Implementation in teacher education and strong leadership at national and municipality levels are key to support successful implementation of movement in Danish schools.

**Conclusion:**

The school system is complex, many factors are important, and these are present within all sectors of the school system. Thus, all parts of the system must take responsibility and engage to succeed.

**Funding:**

This project was supported by Dansk Skoleidræt

